# Synthesis of bismuth oxyhalide (BiOBr _z_I _(1-__z)_) solid solutions for photodegradation of methylene dye

**DOI:** 10.12688/aasopenres.13249.1

**Published:** 2021-08-23

**Authors:** Robert O. Gembo, Ochieng Aoyi, Stephen Majoni, Anita Etale, Sebusi Odisitse, Cecil K. King'ondu

**Affiliations:** 1 Department of Chemical and Forensic Sciences, Botswana International University of Science and Technology, Palapye, Botswana; 2 Department of Chemical, Materials, and Metallurgical Engineering, Botswana International University of Science and Technology, Palapye, Botswana; 3 Global Change Institute, University of the Witwatersrand, Johannesburg, South Africa; 4 Department of Physical Sciences, South Eastern Kenya University, Kitui, Kenya

**Keywords:** Hydrothermal, Flower-like, Bandgap, Thermal effect, Solar, Photocatalysis, Ultraviolet, Superoxide, Photogenerated holes

## Abstract

**Background:** The removal of textile wastes is a priority due to their mutagenic and carcinogenic properties.  In this study, bismuth oxyhalide was used in the removal of methylene blue (MB) which is a textile waste. The main objective of this study was to develop and investigate the applicability of a bismuth oxyhalide (BiOBr _z_I _(1-z)_) solid solutions in the photodegradation of MB under solar and ultraviolet (UV) light irradiation.

**Methods:** Bismuth oxyhalide **(**BiOBr _z_I _(1-z)_) (0 ≤ z ≤ 1) materials were successfully prepared through the hydrothermal method. Brunauer-Emmett-Teller (BET), transmission electron microscope (TEM), X-ray diffractometer (XRD), and scanning electron microscope (SEM) were used to determine the surface area, microstructure, crystal structure, and morphology of the resultant products. The photocatalytic performance of BiOBr _z_I _(1-z)_ materials was examined through methylene blue (MB) degradation under UV light and solar irradiation.

**Results:** The XRD showed that BiOBr _z_I _(1-z) _materials crystallized into a tetragonal crystal structure with (102) peak slightly shifting to lower diffraction angle with an increase in the amount of iodide (I ^-^). BiOBr _0.6_I _0.4 _materials showed a point of zero charge of 5.29 and presented the highest photocatalytic activity in the removal of MB with 99% and 88% efficiency under solar and UV irradiation, respectively. The kinetics studies of MB removal by BiOBr _z_I _(1-z) _materials showed that the degradation process followed nonlinear pseudo-first-order model indicating that the removal of MB depends on the population of the adsorption sites. Trapping experiments confirmed that photogenerated holes (h ^+^) and superoxide radicals ( ^•^O _2_^−^) are the key species responsible for the degradation of MB.

**Conclusions:** This study shows that bismuth oxyhalide materials are very active in the degradation of methylene blue dye using sunlight and thus they have great potential in safeguarding public health and the environment from the dye’s degradation standpoint. Moreover, the experimental results agree with nonlinear fitting.

## Introduction

The availability of clean water is key for human and environmental health. The rise in demand for dyed commodities such as plastics, and textiles has led to an increase in the discharge of organic dyes into the environment and the deterioration of water quality. Approximately 17–20% ^1, 2^ of water pollution is from the dyeing and textile industries. About 7×10 ^5^ tons ^3– 5^ of organic dyes are used to manufacture 3×10 ^7^ tons of textiles annually ^3^. Textile dyeing also consumes large volumes of water. However, approximately 30%, (2 × 10 ^5^ tons) ^4^, of dyes are lost as waste during the manufacturing process thereby finding their way into the environment and water bodies.

Therefore, much attention has been directed towards developing efficient treatment techniques for these organic dyes in wastewater in the recent past. These techniques include conventional ones such as biological, physical, and chemical treatment ^5^. However, several limitations associated with these methods have been reported. First, treated effluent often does not meet the set standards for parameters such as color and chemical oxygen demand (COD) due to the limited effectiveness of these methods in breaking down the dyes. Second, the wastewater often contains both inorganic and organic compounds, making it hard to treat by conventional methods ^3, 6– 8^. Therefore, there is a need to employ a method that is effective, less costly, and environmentally friendly. Oxidative processes such as photocatalysis, the Fenton method, photolysis, sonolysis, sonocatalysis, sonoFenton, photo-Fenton, and ozonolysis ^6, 9– 17^ have recently been explored. Among these, photocatalysis, which depends on *in-situ* photogenerated positively charged holes (h ^+^), hydroxyl radicals ( ^•^OH), negatively charged electrons (e−), superoxide radicals ( ^•^O _2_^−^) has been demonstrated to be promising in terms of cost, toxicity, recyclability, mild reaction conditions, ease of operation, efficiency, and high degradation ability ^18– 23^. Photocatalysis is also a better option because it could potentially mineralize bio-recalcitrant compounds to produce carbon dioxide, water, and other inorganic substances hence no waste is left for secondary disposal ^8^. Various semiconductor photocatalysts such as metal oxides and sulfides have been widely probed and applied in environmental remediation.

Traditionally, TiO _2_ has been known to be effective in the treatment of wastewaters. However, titanium is costly and not readily available. Furthermore, TiO _2_ only absorbs in the ultraviolet (UV) region, which is about 3–5% of the solar spectrum due to its wide bandgap of 3.2 eV ^17, 19, 22^. Instability and photo-corrosion are also drawbacks associated with metal-oxide semiconductors. Much of the inexpensive and abundant visible radiation energy is not harnessed in wastewater treatment with these types of metal oxide photocatalysts because they are only active within the UV region which constitutes a very small fraction of the solar insolation ^18, 24– 27^. Metal sulfides like CoS _2_, In _2_S _3_, CdS, and Sb _2_S _3_ have been studied and found to have a proper location of conduction valence bands and high sensitivity to visible light, however, they are costly, prone to photo-corrosion, and the heavy metals involved are toxic ^2, 19^.

To overcome these drawbacks, it is imperative to develop alternative photocatalytic materials that are more stable but less costly. Bismuth oxyhalide (BiOZ (Z = Cl, Br, I)) materials are a novel class of photocatalyst due to their environmental friendliness, outstanding photocatalytic performance under both solar and UV irradiation, unique optical and electrical properties, unique layered crystal structure, high oxidation capacity, good chemical stability, and internal electric field effect (IEE) ^19, 23, 28– 30^. BiOZ compounds are tetragonal matlockite (space group p4/nmm) which consist of (Bi _2_O _2_) ^2+^ layer interleaved (Z _2_) ^2^ halogen ions (Z = Cl, Br, I) slabs which crystallize into a layered structure, forming [Z–Bi–O–O–Bi–Z] stacked slices. These slices interact with halogen ions along the c-axis via the Weak Van der Waals forces. Due to the layered structure, bismuth oxyhalide compounds, display unique optical, mechanical, and electrical qualities and have found application in fields including photocatalysis, organic synthesis, nitrogen fixation, and solar-driven H _2_ generation. Furthermore, the electric field that forms between (Bi _2_O _2_) ^2+^ and 2Z ^−^ layers enhance the separation of photoexcited e⁻ and h ^+^, thus improving the photocatalytic activity ^30– 32^. Bismuth oxyhalides have been studied as photocatalysts, interfaced with other photocatalytically active materials, and quaternary alloys ^12^.

The yellow colour of BiOBr and the coral red colour of BiOI indicate that they strongly absorb light within the visible range, however, their performance is still low ^8^. Therefore, improving the photocatalytic abilities of BiOZ compounds is necessary for practical applications. Several approaches, for instance, doping with metals and/or non-metals, compositing BiOZ with other materials, for example, TiO _2_, Ag, AgCl, AgBr, WO _3_, and AgPO _4_, noble metals deposition, synthesis of different heterojunctions ^20– 22^ have been shown to enhance the activity of these BiOZ photocatalysts in dye degradation. Zhang *et al.,* synthesized nitrogen-doped graphene quantum dots/BiOZ (Z = Br, Cl) for the removal of rhodamine B (RhB) dye ^14^. Lee *et al.,* prepared AgX (X = Cl, Br, I)/BiOZ for the removal of methyl orange, RhB, and MB ^15^. Qu *et al.,* reported the treatment of methyl orange with TiO _2_/CQDs/BiOZ (Z = Cl, Br, I) heterostructure. It has been shown that the preparation of solid solutions generally enhances the activity of a catalyst.

The development of solid solution has been acknowledged to change the bandgap, crystal, and electronic structures of a photocatalyst which can impact their photocatalytic properties ^16^. Solid solutions such as BiOI _z_Br _1-z_, BiOCl _z_I _1-z_, zBiOBr-(1-z)BiOI, BiOBr _z_I _1-z_, and BiO(ClBr) _1-z_/2I _z_ have been synthesized and have shown improved photocatalytic activity when compared to corresponding single components ^19, 23, 29, 31^. These materials have been utilized in the remediation of wastewater containing different organic dyes. Gnayem *et al.,* ^20^ prepared hierarchical nanostructured 3D flowerlike BiOCl _z_Br _1−z_ for RhB degradation under visible light irradiation. Zhang *et al.,* ^16^ fabricated BiOBr _z_I _1−z_ nanoplates for the removal of RhB. Zhang *et al.,* ^21^ synthesized BiOCl _z_Br _1-z_ nanoplate solid solutions for the removal of RhB under the irradiation from visible light. Zhang *et al.,* ^22^ synthesized BiOBr _z_I _1-z_ solid solutions for the removal of various dyes from textile wastes.

However, to the best of the authors’ knowledge, the application of BiOBr _z_I _(1-z)_ solid solutions in the treatment of methylene blue (MB) dye and comparative studies on the photocatalytic performance of these materials under natural sunlight and UV light has not been reported. Additionally, no report on the comparison of the experimental kinetic data to both linear and non-linear fitting using Langmuir-Hinshelwood (L-H) kinetic model. Methylene blue dye is extensively used in printing and textile dyeing industries due to its intense blue colour and thus a common pollutant in industrial wastewaters. The purpose of the present study is to enhance the absorption of visible light by BiOZ. Thus, for the improved visible-light uptake, the BiOBr _z_I _(1-z)_ solid solutions with a suitable bandgap were prepared by doping the bromide and the iodide ions through varying the amounts of the dopants from 0 to 1 in the compound. The photocatalytic activity of BiOBr _z_I _(1-z)_ solid solutions was evaluated by determining their efficiency in removing MB under solar and UV light exposure. Furthermore, the efficiency of the most active materials was optimized by careful adjustment of catalyst dosage, temperature, and pH, while the degradation pathway was examined by use of different scavengers.

## Methods

### Duration of methodology

The preparation of the photocatalysts was done between Oct–Nov 2018. After the preparation, the degradation efficacy of the prepared samples was tested before characterization. This was carried out in Jan 2019. The XRD, SEM, TEM, BET, Raman characterization was carried out between Feb–Dec 2019. The photodegradation studies were carried out throughout 2019 and between Jan–May 2020. The XRD and Raman analysis was carried out at the Botswana International University of Science and Technology (BIUST). The SEM characterization was done at Botswana Institute for Technology Research and Innovation (BITRI), while TEM and BET analysis was done at Global Change Institute, University of the Witwatersrand, South Africa. No adjustment was done to the scanning electron microscope micrographs. All the degradation performance was carried out at BIUST. OriginPro 8.5.0 SR1 b161 software was used for data analysis and graphing. Alternatively, R and excel can be applied for analysis. 

### Chemicals and materials

Analytical standard bismuth nitrate (Bi(NO _3_) _3_.5H _2_O, Cat No. 10035-06-0), potassium iodide (KI, Cat No. 290813PQ), sodium bromide (NaBr, Cat No. 27656), acetic acid (CH _3_COOH, Cat No. AA539), ethylenediaminetetraacetic acid disodium (EDTA, Cat No. 1046725) salt, sodium nitrate (NaNO _3_, Cat No. 030311SN), sodium hydroxide (Cat No. 051214SO), silver nitrate (Cat No. 111214SN), benzoquinone (Cat No. 606-013-00-3), tert-butanol (Cat No. 603-005-1), nitric acid (Cat No. 110815NC) and methylene blue (MB, Cat No. MB057) reagents used in this work were acquired from Sigma-Aldrich. The dye working solutions used in this investigation were made by adding a 10 mg methylene blue in 1000 mL of distilled water (DI) to make the desired concentrations.

### Synthesis of BiOBr _z_I _(1-z)_ solid solution

Bismuth oxyhalide (BiOBr _z_I _(1-z)_) solid solutions were synthesized via hydrothermal method at 160 ℃. Typically, a 0.4123 mM Bi(NO _3_) _3_.5H _2_O solution was prepared in CH _3_COOH. A solution mixture having stoichiometric amounts of KI and NaBr was then dropwisely added into the above Bi-based solution while continuously stirring. The resultant mixture was then put into 23 mL stainless steel autoclave lined with teflon (4749 PTFE A280AC Teflon, Parr Instrument Company) and heated at 160 ℃ in an oven, for 24 h. When the reaction was complete, the sample was naturally cooled to room temperature, centrifuged (Heraeus Megafuge 40 centrifuge) 6 times at 6000 rpm to effectively separate the product, and washed 6 times with deionized. The product obtained was then dried at 70 ℃ for 24 h and used in photodegradation reactions. The molar ratios of Br ^-^ and I ^-^ was varied between 0 and 1 with Z = 0.0, 0.2, 0.4, 0.6, 0.8, 1.0 for BiOBr _z_I _(1-z)_ materials and samples prepared hereafter labelled BiOI, BiOBr _0.2_I _0.8_, BiOBr _0.4_I _0.6_, BiOBr _0.6_I _0.4_, BiOBr _0.8_I _0.2_, and BiOBr, respectively. 

### Point of zero charge (pH _PZC_) determination

The procedure used in this work was adopted from Tahira *et al.* ^23^. The PZC values for BiOBr _z_I _(1-z)_ samples were determined in 0.1 M solution of NaNO _3_ at 298 K. Typically, 0.1 g of the samples was dispersed in NaNO _3_ solution (0.1 M, 30 mL) in various reaction flasks. Adjusting the initial pH of the mixtures to 2, 3, 4, 5, 6, 7, 8, 9, 10, and 11 was done using 0.1 M HNO _3_ and NaOH. Each reaction vessel was then agitated at 130 rpm in a shaker (Stuart orbital shaker SSL1) for 24 h. The final pH of the mixtures was determined by a Basic20 pH-meter (Crison). A graph of the difference between final and initial pH (ΔpH) values was then plotted against the initial pH values. The pH _PZC _ was taken to be the initial pH at which ΔpH is 0.

### Characterization

The crystal structure of the synthesized materials was obtained by X-ray diffraction (XRD) at room temperature by a Bruker D8 Advance powder diffractometer with a Cu tube X-ray source and a LynxEye XE-T energy-dispersive strip detector. The radiation used was a Cu-Kα (λ = 1.54056 nm) with increasing current and voltage of 40 kV and 40 mA, correspondingly. The patterns were acquired at a 2θ scan rate of steps 0.02 degrees with a time of 0.500 sec/step from 5 to 90°. Raman spectra were acquired by LabRAM HR800 Raman spectrophotometer, exciting the samples with 532 nm laser. FEI Tecnai G2 Spirit transmission electron microscope at an acceleration voltage of 120 kV and scanning electron microscope (SEM, Gemini SEM 500, Carl Zeiss, 5.00 KX magnifcation) with energy-dispersive X-ray detector (EDX) were applied to obtain the morphology and the elemental composition of the BiOBr _z_I _(1-z)_ solid solutions. Porosity and surface area investigations were performed by Brunauer-Emmett-Teller technique (Micro metrics Tristar 3000 porosity analyzer). The samples were degassed at 150℃ for 5 h before sample analysis at liquid nitrogen temperature (77.350 K). Ultraviolet-visible (UV-Vis) spectrophotometer (UV201, Shimadzu) was applied to study the photodegradation of MB.

### Photodegradation studies

To test for the photodegradation ability of the BiOBr _z_I _(1-z)_ solid solutions, 50 mg of the material was added in 50 mL of 10 mgLˉ ^1^ dye solution. The equilibrium (adsorption-desorption) between the photocatalyst and the solution containing the dye was attained through the stirring of the mixture in the dark for 30 min, thereafter the photodegradation was carried out under solar irradiation and UV lamp operating at 0.16 Amps with a UV output of 254 nm (UVP UVG-54). The set up for the photodegradation under UV lamp and solar is shown in Figure S1. During the reaction process, 2 mL of aliquot was drawn at 30-minute intervals from the mixture to monitor the degradation of MB. The separation of the photocatalyst and the dye solution was achieved through centrifuging the aliquot for 7 min at 13000 rpm. The concentration was monitored using UV-Vis spectrophotometer within a 200–800 nm range.

The absorption of MB was examined at a maximum wavelength of ~661 nm, according to the Beer-Lambert law. An experiment without a sample (blank) was also carried out under UV and solar irradiation as a control. The same procedure was followed for the two conditions (solar and UV light). The active species responsible for the photodegradation process was investigated by conducting scavenging experiments. Tert-butanol (TBA), EDTA, silver nitrate, (AgNO _3_), and P-benzoquinone (BQ) were applied to quench ^•^OH, h ^+^, e⁻, and ^•^O _2_^−^, respectively. The concentration of the quenchers used was 1 mM.

## Results and discussion

### X-ray diffraction

**Figure 1.**
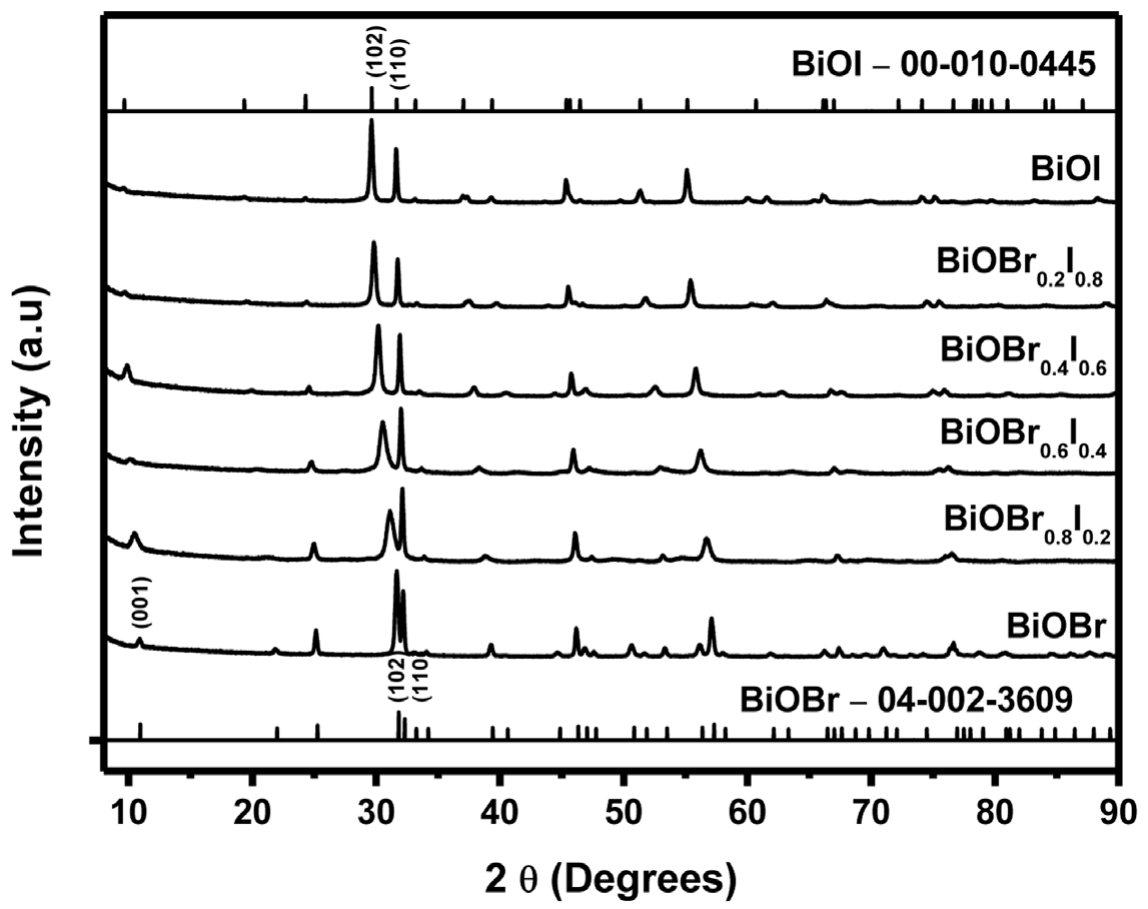
X-ray diffraction (XRD) patterns of BiOBr _z_I _(1-z)_ materials.

Figure 1 displays the XRD spectra of BiOI, BiOBr _0.2_I _0.8_, BiOBr _0.4_I _0.6_, BiOBr _0.6_I _0.4, _BiOBr _0.8_I _0.2_, and BiOBr.

It was observed that all the diffraction peaks of the spectra are sharp showing an efficacious crystallization of BiOBr _z_I _(1-z)_ materials synthesized via the hydrothermal method. The pure BiOBr _z_I _(1-z)_ materials can be attributed to tetragonal structure BiOBr (PDF Card No. 04-002-3609) and BiOI (PDF Card No. 00-10-0445). Even though the composition of the materials is different, they possess a similar tetragonal phase. The BiOBr peaks at 2θ = 11.0, 31.8, and 32.3º are assigned to the hkl values (001), (102), and (110), respectively. While the BiOI peaks at 2θ = 29.6 and 31.7º can be respectively assigned to the hkl values (102) and (110).

There is an observable shift in the diffraction peaks to lower diffraction angles with an increase of I content in BiOBr _z_I _(1−z)_ compound. This observation was also reported by Xu *et al.,* ^19^ who suggested that the shift is a result of bromide ions substitution (ionic radius of 0.196 nm) with the larger iodide ions (ionic radius of 0.216 nm) that leads to an expansion of the interlayer spacing. The percentage difference between the ionic radii of bromide and iodide ion is 9%, a value less than the maximum of 15% for substitution to occur. Additionally, the unlimited formation of solid solutions between BiOI and BiOBr can further be shown by the shift in diffraction peaks to lower angles as the amount of iodine increases. ^25^. Cell parameters also increased gradually with an increase in I content (Extended data, Table S1 ^33^) ^18^. Figure 1 shows a perfect change from BiOBr to BiOI in the BiOBr _z_I _(1-z)_ pattern, this has been observed before by Lei *et al.* ^26^. There are no peaks attributed to impurities, indicating that the samples consisted purely of the desired phases.

### Raman spectroscopy

To further understand the structure of the as-prepared materials, the Raman technique was used. Figure S2 (Extended data ^33^) represents the Raman spectra of BiOBr _z_I _(1-z)_ solid solutions prepared by varying amounts of Br ^-^ and I ^- ^ions. To show a variation in structural features of BiOBr _z_I _(1-z)_ solid solutions, the Raman spectra were recorded from 25 – 500 cm ^–1^. Bismuth oxyhalide belongs to the tetragonal PbFCI type structure with space of p4/nm, hence the active modes of the Raman exhibited are A _1_g, Eg, and B _1_g. BiOBr samples presented two major Raman peaks at 45 and 106 cm ^–1^ allotted to the first-order vibration of Bi-O and A _1_g internal Bi–Br stretching mode, respectively ^27^. The stronger bands of BiOI at 78 and 145 cm ^–1 ^is allotted to the A _1_g stretching mode from internal Bi-I bonds. It can also be observed that the peak at 45 cm ^−1 ^diminishes while the peak 106 cm ^−1^ changes to a lower value of 78 cm ^–1 ^as the amount of Iˉ ions increases. The shifting of the peaks to the lower Raman value agrees with the XRD results, which also indicates that as the amount of Iˉ increases the (102) plane shifts to the lower angles.

### Morphological analysis by SEM and TEM

**Figure 2.**
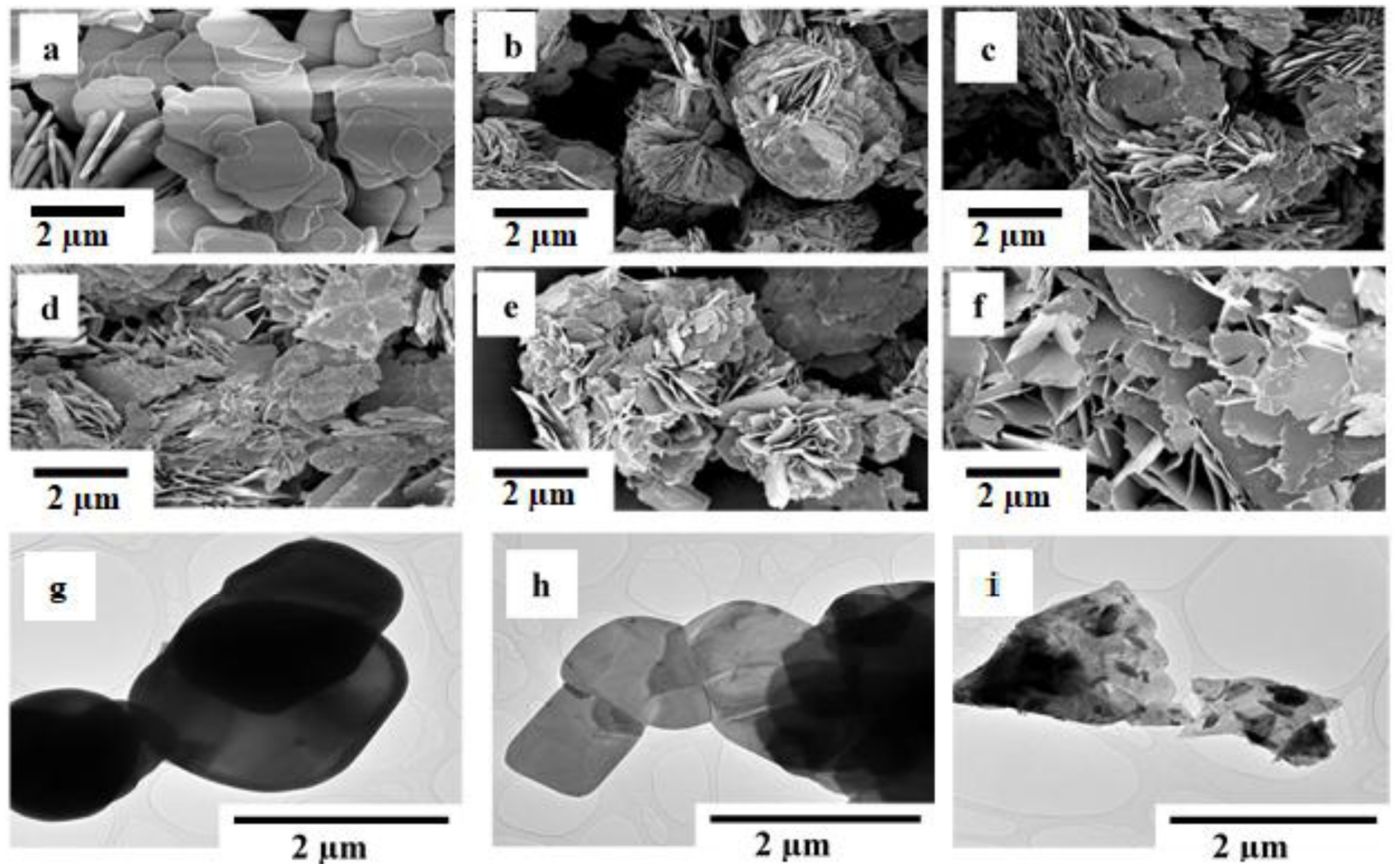
SEM micrographs of ( **a**) BiOBr, ( **b**) BiOBr _0.8_I _0.2_, ( **c**) BiOBr _0.6_I _0.2_, ( **d**) BiOBr _0.4_I _0.6_, ( **e**) BiOBr _0.2_I _0.8_, ( **f**) BiOI. TEM images of ( **g**) BiOBr, ( **h**) BiOBr _0.6_I _0.4_, and ( **i**) BiOI. No modifications have been made to the images apart from adding the scales.

The examination of surface morphologies of BiOBr _z_I _(1-z)_ solid solutions was done using SEM, as shown in Figure 2(a–f). The samples display plate-like morphology with interleaved nanoplates of varying sizes. The formation of the plate-like morphology can be attributed to the internal structure of bismuth oxyhalide (BiOZ, Z = Cl, Br, I) where (Bi _2_O _2_) ^2+^ layers sandwich between the two slabs of halogen atoms resulting in the anisotropic growth of BiOZ at a c axis forming 2D structures. As shown in Figure 2a, pure BiOBr formed relatively large plates which upon iodine doping reduced in size and self-assembled in 3D flowers, Figure 2b,c. As the amount of bromide decreased and iodine increased towards pure BiOI, the size of the flakes increased forming relatively large 2D structures akin to those of pure BiOBr. TEM images were obtained to further understand the BiOBr, BiOBr _0.6_I _0.4_, and BiOI SEM morphology. The TEM micrographs, Figure 2g, h, i, confirm the plate-like morphology observed by SEM for all the BiOBr _z_I _(1-z)_ materials. The presence of Br, Bi, O, and I was confirmed by EDX examination. The amounts of the elements detected agree with the BiOBr _z_I _(1-z)_ formula, (Extended data, Figure S3 ^33^).

### Surface area analysis

The textural properties of BiOBr, BiOBr _0.6_I _0.4_, and BiOI materials were obtained using N _2_ adsorption-desorption analysis. The isotherms displayed in Figure S4a (Extended data ^33^) can be allotted to type III based on IUPAC classification. This type of isotherm has no recognizable monolayer formed due to the relatively weak interaction between the adsorbent and the adsorbate hence leading to a clustering of adsorbed molecules around the most favourable sites on the surface of a macroporous or nonporous solid. Compared to type II isotherms, the quantity of molecules adsorbed remains finite at p/p0 = 1 which is the saturation pressure ^28^. The BET surface areas of the as-prepared materials were 0.517, 3.249, and 1.890 m ^2^/g for BiOBr, BiOBr _0.6_I _0.4_, and BiOI, respectively. The BiOBr sample has a larger and thicker microplate, which has a much lower surface area. Figure S4b (Extended data ^33^) shows Barret-Joyner-Halenda (BJH) plots of BiOBr, BiOBr _0.6_I _0.4_, and BiOI materials obtained from desorption isotherms. The BJH plots confirmed that the corresponding macropores peaks of the pore-diameters distributions of BiOBr, BiOBr _0.6_I _0.4_, and BiOI could be found up to 159, 170, and 153 nm, respectively. The BJH result shows that the pore diameter distributions of the materials are wide. This can be ascribed to the inter-crossing of the spaces between the microplate structure ^29^.

### pH _PZC_ and optical properties

The results are displayed in Figure S5 of the Extended data ^33^. The PZC (the pH at which the surface charge of a material is 0) of BiOBr, BiOBr _0.8_I _0.2_, BiOBr _0.6_I _0.4_, BiOBr _0.4_I _0.6_, BiOBr _0.2_I _0.8_, and BiOI materials was found to be 5.27, 5.27, 5.29, 5.31, 5.38, and 5.39, respectively. At a pH lower than the PZC, the material will be positively charged whereas at a pH higher than PZC the materials acquire a negative charge ^30^. The photocatalytic performance of a semiconductor depends on the band structure of the photocatalyst. Figure 4 shows the UV/Vis spectra of the as-synthesized BiOBr _z_I _(1-z)_ materials.

**Figure 3.**
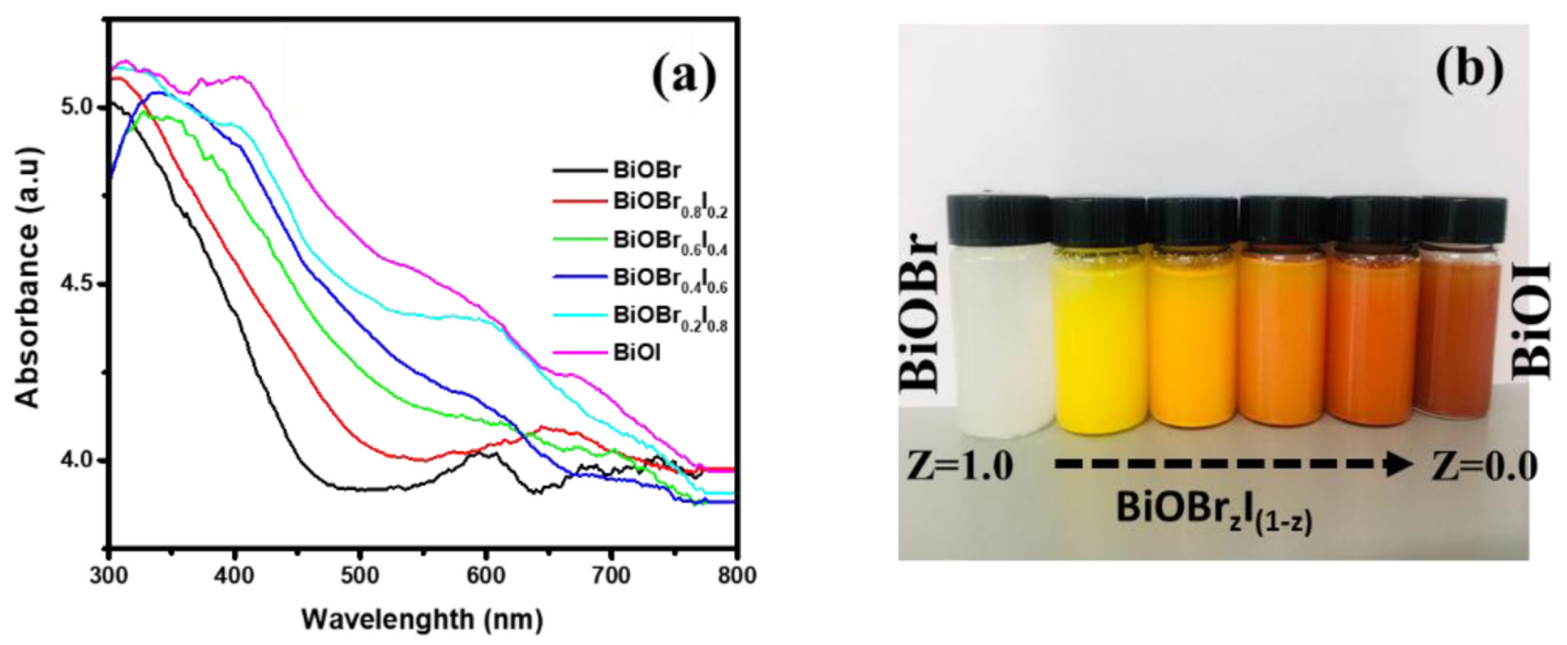
( **a**) Ultraviolet-visible absorption spectra for the BiOBr _z_I _(1-z)_ solid solutions ( **b**) optical images of BiOBr _z_I _(1-z) _ suspension in methanol.

When the composition of iodine is increased from 0 to 1, there is a significant redshift of the absorption edges from 478 to 632 nm for pure BiOBr and BiOI, respectively. Whereas the absorption edges of BiOBr _z_I _(1-z)_ materials whose composition lie between that of BiOBr and BiOI were found to be 546, 577, 591, and 611 nm for Z = 0.8, 0.6, 0.4, and 0.2, respectively. The change is consistent with the colour of the material, from white to coral-red ( Figure 3b). The spectra revealed that the absorption of BiOBr is slightly within the visible region and the absorption edge of BiOI extends towards the visible region indicating that it is highly responsive within visible light. The bandgap energy (E _g_) values of the obtained BiOBr _z_I _(1-z)_ materials are determined using Equation 1 below:


$$\alpha hv=A(hv-E_{g})n/2\;(1)$$

**Figure 4.**
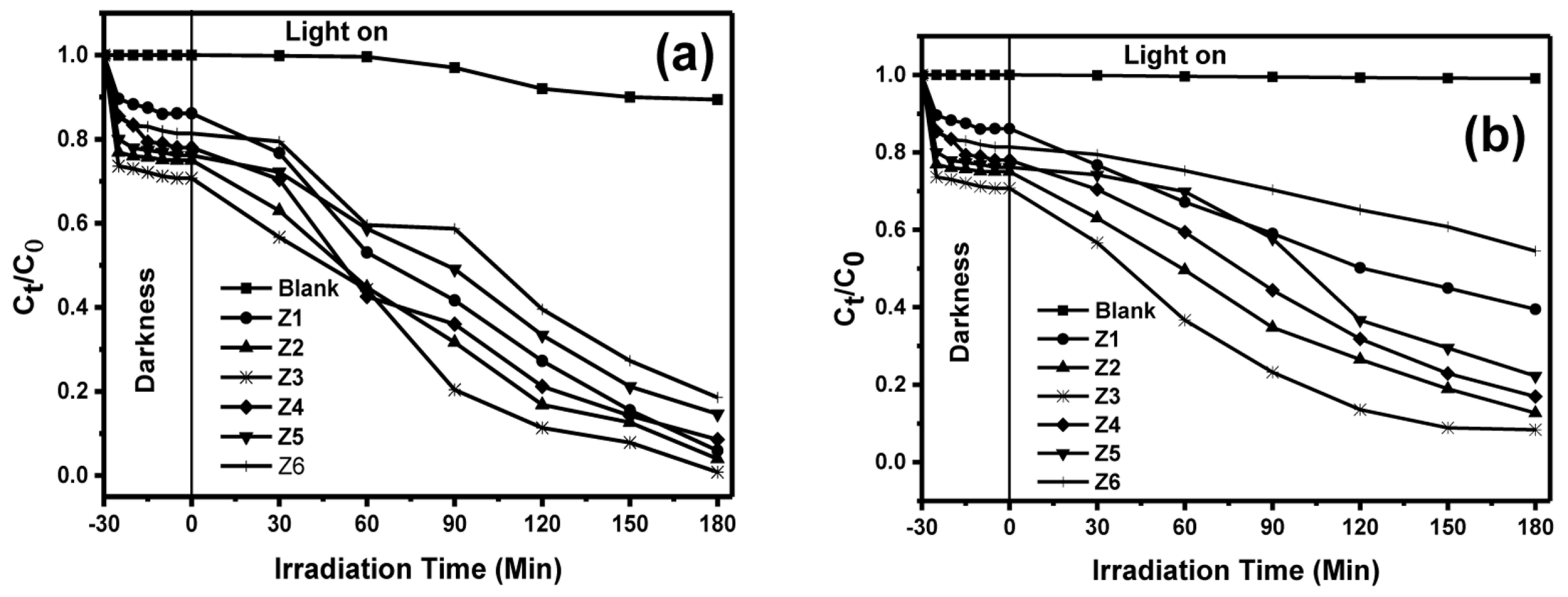
Photodegradation efficiencies under ( **a**) solar and ( **b**) ultraviolet (UV) light after 3 h of irradiation.

where α is absorption coefficient, h is Plancks constant, ν is frequency of light, A is absorbance, and E _g_ is bandgap energy ^8^, and n is dependent on the transition characteristics of the semiconductor. For BiOZ, n is 4 owing to its indirect change. The corresponding bandgap energies of BiOBr _z_I _(1-z)_ materials were calculated and the result is shown in Table 1. The bandgap energy of the materials could be tailored from 2.59 to 1.96 eV by reducing the value of Z from 1 to 0 as shown in Table 1, demonstrating that doping I atoms into BiOBr crystal reduced the bandgap and increased the absorption range of BiOBr. This improves the photocatalytic performance of BiOZ.

**Table 1.** Absorption thresholds, electronegativity, bandgap energy, the conduction band (CB) and valence band (VB) edges of as-prepared BiOBr _z_I _(1-z)_ materials.

Parameters	BiOBr _z_I _(1-z)_ materials					
	Z = 1.0	Z = 0.8	Z = 0.6	Z = 0.4	Z = 0.2	Z = 0.0
Absorption thresholds (nm)	478	546	577	591	611	632
Electronegativity (χ) (eV)	6.18	6.14	6.08	6.04	5.99	5.94
E _g_ (eV)	2.59	2.27	2.15	2.10	2.03	1.96
E _CB_ (eV)	0.39	0.51	0.51	0.49	0.48	0.46
E _VB_ (eV)	2.98	2.78	2.66	2.59	2.51	2.42

The Mulliken electronegativity theory was used in calculating the conduction band (CB) and valence band (VB) electric potentials using Equation 2 and Equation 3:


$$E_{CB}=\chi -E^{c}-\frac{1}{2}E_{g}\;(2)$$

$${\text{E}}_{\text{VB}}={\text{E}}_{\text{CB}}+{\text{E}}_{\text{g}}\;(3)$$

where E _CB_ and E _VB_ are the electric potentials edges, respectively. E ^c^ is the free electrons energy on the hydrogen scale (4.5 eV), E _g_ is semiconductor’s bandgap energy. While χ is the semiconductor electronegativity, equivalent to geometric mean electronegativity of atoms forming the compound.

From the calculations obtained from the UV-Vis results, the CB and VB of BiOBr _z_I _(1-z)_ materials were approximated and the results are presented in Table 1. The gradual decrease in VB edge potential from 2.98 to 2.42 with increasing iodine composition indicates a weak oxidation ability and a stronger light absorption capability ^31^. The generation of ^•^O _2_^−^ radicals depends on the CB edge potential, the more positive the CB, the more difficult it is to generate ^•^O _2_^−^ radicals. Moreover, increased iodide concentration leads to a reduction in the level of VB. This in turn reduces the bandgaps energy thereby facilitating photosensitization of the catalyst. Therefore, the highest photocatalytic performance of BiOBr _0.6_I _0.4_ material; the most active material, is ascribed to the suitable bandgap structure that may attain the equilibrium between the light absorption capacity and redox power.

### Photodegradation studies

***Adsorption of MB by BiOBr _z_I _(1-z)_*.** The adsorption ability of a photocatalyst is well-known to play an important role in the photodegradation process. Figure S6 in the Extended data ^33^ displays the MB adsorption uptakes by BiOBr _z_I _(1-z)_ materials at constant concentration and catalyst dosage. The adsorption uptake values for the MB dye by BiOBr, BiOBr _0.8_I _0.2_, BiOBr _0.6_I _0.4_, BiOBr _0.4_I _0.6_, BiOBr _0.2_I _0.8_, and BiOI were 12, 17, 19, 16, 15, and 14%, respectively. The adsorption efficiency was found to be in the order BiOBr _0.6_I _0.4 _> BiOBr0 _.8_I _0.2 _> BiOBr _0.4_I _0.6 _> BiOBr _0.2_I _0.8 _> BiOI > BiOBr. Fitting of the adsorption data to adsorption isotherms using nonlinear model fitting procedures (Extended data, Figure S7 ^33^) indicated that the process of adsorption was according to Langmuir. The Langmuir constant and the maximum adsorption capacity of BiOBr _0.6_I _0.4_ were found to be 6.10×10 ^‒2^ L/mg and 31.5 mg/g, respectively. This implies that at the highest concentration used in this study, the adsorption sites were not saturated with the dye.

***Kinetics, scavenging experiments and recyclability*.** To broadly understand the photocatalytic ability of the as-prepared materials, the removal of MB was performed under irradiation from solar and UV light in the presence and absence of the photocatalyst materials. Stirring of photocatalyst and dye mixture was carried out in the dark for half an hour before illumination to attain adsorption-desorption equilibrium (Extended data, Figure S6 ^33^). Figure 4a, b shows the removal of MB under the irradiation from solar and UV light, respectively. The degradation in the absence of the photocatalyst (photolysis) both under the solar and UV light showed a negligible change indicating that MB is stable under both light sources hence photolysis can be neglected. Therefore, the removal of MB can be attributed to solar and UV light in the presence of the photocatalysts. The photodegradation efficiency was calculated using Equation 4 below:


$$\mathrm{Degradation}\;\mathrm{efficiency},\;\mu =\frac{C_{0}-C_{t}}{C_{0}}x100\%\;(4)$$

where C _0_ is the concentration of MB dye at equilibrium, C _t_ the concentration at the time, interval, t. Table S2 in the Extended data ^33^ shows the percent degradation of MB after 3 h under solar and UV-light irradiation using BiOBr, BiOBr _0.8_I _0.2_, BiOBr _0.6_I _0.4_, BiOBr _0.4_I _0.6_, BiOBr _0.2_I _0.8,_ and BiOI. BiOI consistently displayed the least photocatalytic performance amongst all the materials despite absorbing at the longest wavelength of 632 nm. This could be due to a higher recombination rate of photoexcited e⁻ and h ^+^ pair ^8^. Photocatalytic ability in most cases depends on the surface area, such that the lower the surface area, the lower the activity due to lower adsorption of the dye solution. However, despite their low surface areas, BiOBr _z_I _(1-z)_ solid solutions in our work showed high photocatalytic activity revealing that the speciﬁc surface area was not the key reason for the improved photocatalytic performance of this material. The higher activity could be attributed to the electric field that built-in between (Bi _2_O _2_) ^2+^ and 2Z ^−^ layers enhancing the separation of photoexcited e⁻ and h ^+^, thus improving the photocatalytic performance.

Importantly, although no obvious trend in the photocatalytic activity of the obtained materials was observed, doping seemed to improve the photocatalytic ability of the photocatalysts. The bandgap of BiOBr _z_I _(1-z)_ solid solutions ranges from 1.96 to 2.59 eV which is generally low, hence requires lower energy for activation. After 3 h of irradiation both under solar and UV light, BiOBr _0.6_I _0.4_ recorded the highest activity with degradation efficiency of 99% and 88% under solar and UV-light, respectively. The greatest photocatalytic performance of BiOBr _0.6_I _0.4_ can be linked to its flower-like morphology that could lead to stronger absorption of light than the 2D structures. Finally, the optimal composition of BiOBr _0.6_I _0.4_ promotes the photoactivity by minimizing the recombination of photoexcited electron-hole pair.

The model that was used to fit the experimental data obtained from UV-light irradiation was pseudo-first-order. The equation ln (C _o_/C _t_) = κt, was used to determine the pseudo-first-order rate constant. In the equation, ln (C _o_/C _t_) = κt, κ and t denote the rate constant (min ^−1^) and time of irradiation, respectively. C _o_ is the initial concentration of MB dye, whereas C _t_ is the concentration at the time interval, t. As presented in Table S3 of the Extended data ^33^ the reaction rate constants of blank, BiOI, BiOBr _0.2_I _0.8_, BiOBr _0.4_I _0.6_, BiOBr _0.6_I _0.4_, BiOBr _0.8_I _0.2_, and BiOBr were 5.56 x 10 ^-5^, 0.00432, 0.00846, 0.01188, 0.00696, 0.00505, 0.00192, and min ^-1^, respectively. The reaction rate constants results indicate that BiOBr _0.6_I _0.4_ with the highest reaction rate of 0.01188 min ^-1 ^was approximated to be three and six times greater than BiOBr and BiOI, respectively. 

**Figure 5.**
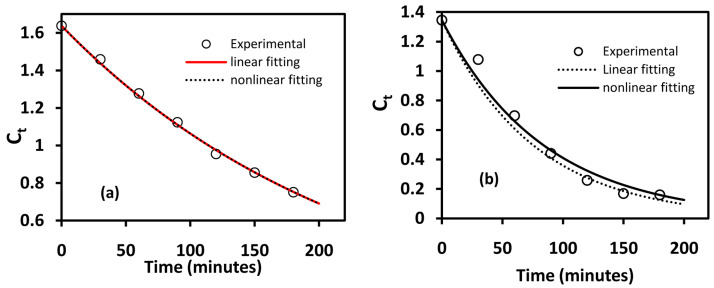
Linear and nonlinear fitting of ( **a**) BiOBr and ( **b**) BiOBr _0.6_I _0.4_ to L-H kinetic model.

The pseudo-first-order kinetic model, derived from the Langmuir-Hinshelwood (L-H) kinetic model under conditions of low dye concentration, has been used extensively to evaluate the reaction kinetics of photodegradation processes ^34, 35^. Whereas the linear equation has been widely (almost exclusively) used during kinetic studies in photodegradation studies, it has been noted that the nonlinear model fitting gives more accurate results. This is highlighted in Figure 5, which shows a comparison of nonlinear and linear fitting for BiOBr and BiOBr _0.6_I _0.4_ in which nonlinear model-fitting has a lower sum squared error (SSE) values compared to linear fitting. As the deviation from L-H kinetic model increases, the differences in parameters obtained from nonlinear and linear model fitting becomes significant as shown in Table 2 in which the differences in the obtained rate constants are 1.8% for BiOBr and 10% for BiOBr _0.6_I _0.4_. Results for nonlinear fitting of BiOBr _z_I _(1-z)_ materials are shown in Figure S7 of the Extended data ^33^ and summarised in Table 2.

**Table 2.** Summary of linear and nonlinear fitting parameters for BiOBr and BiOBr _0.6_I _0.4_.

	BiOBr			BiOBr _0.6_I _0.4_		
	Sum squared error (SSE)	R ^2^	*k*/min	Sum squared error (SSE)	R ^2^	*k*/min
Nonlinear	0.0012	0.9971	0.00432	0.028	0.9627	0.01188
Linear	0.04	0.9827	0.0044	0.14	0.9556	0.01322

**Figure 6.**
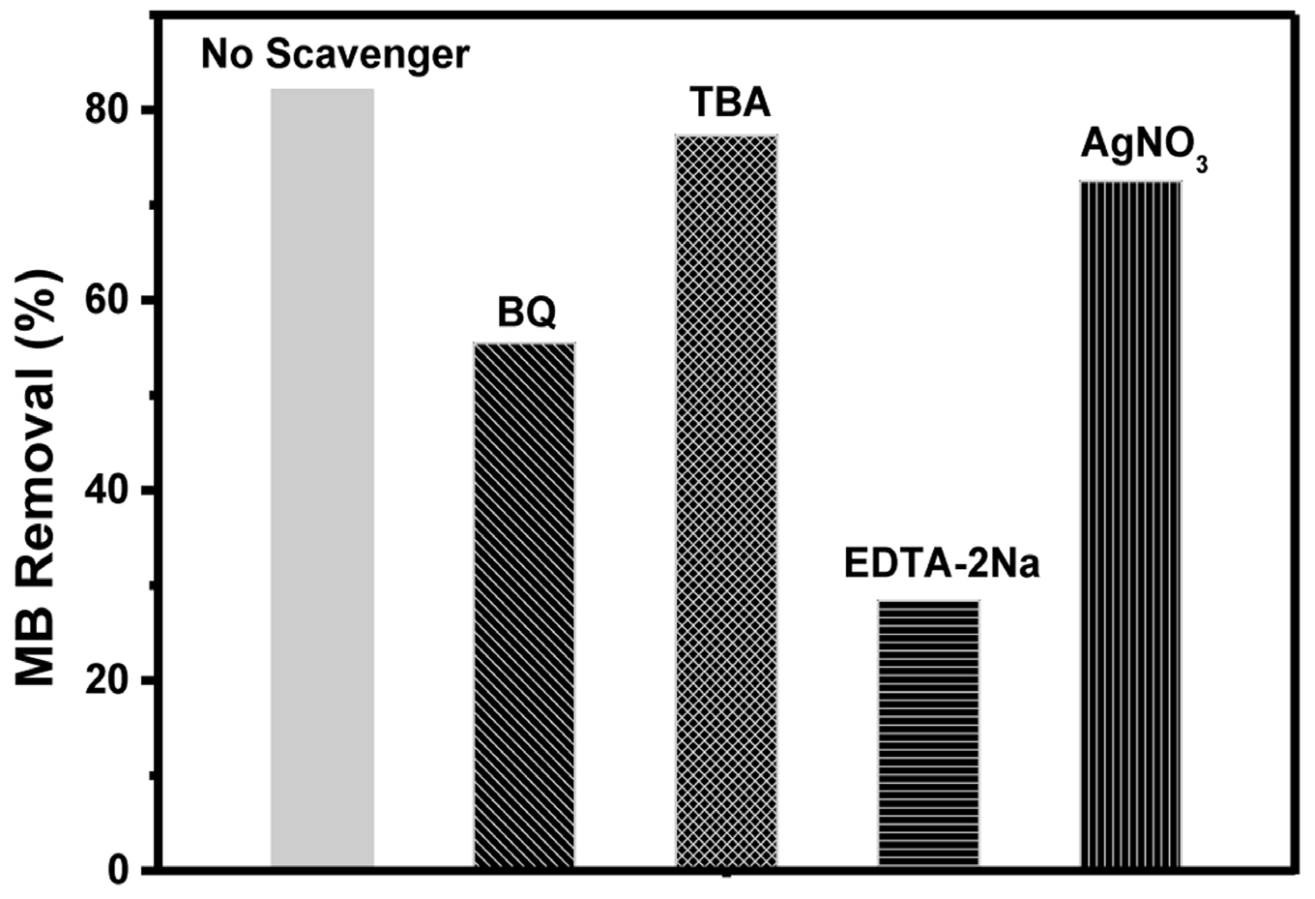
Effects of quenchers on the removal of methylene blue (MB).

The main active species involved during photocatalysis are known to be O _2_^•−^, e ^-^, h ^+^, and HO ^•^. However, the main active species during the degradation of MB was established through the quenching experiment over BiOBr _0.6_I _0.4_ under the irradiation from the UV light. P-benzoquinone (BQ), silver nitrate (AgNO _3_), EDTA-2Na, and tert-butanol (TBA) was added to the reaction vessels to quench O _2_^•−^, e ^−^, h ^+^, and HO ^•^ respectively. When TBA and AgNO _3_ was added, the removal efficiency was insignificant compared to when no quencher was added as presented in Figure 6. This indicates that the HO ^•^ and e ^-^ were not the main active particles in the photodegradation reaction system. However, when BQ and EDTA-2Na were added, there was an obvious reduction in degradation efficiency as a result of the suppression of h ^+^ and O _2_^•−^ which were the major species in the reaction system. Rashid *et al.,* ^36^ made similar conclusions for the photocatalytic mineralization of aqueous ciprofloxacin.

**Figure 7.**
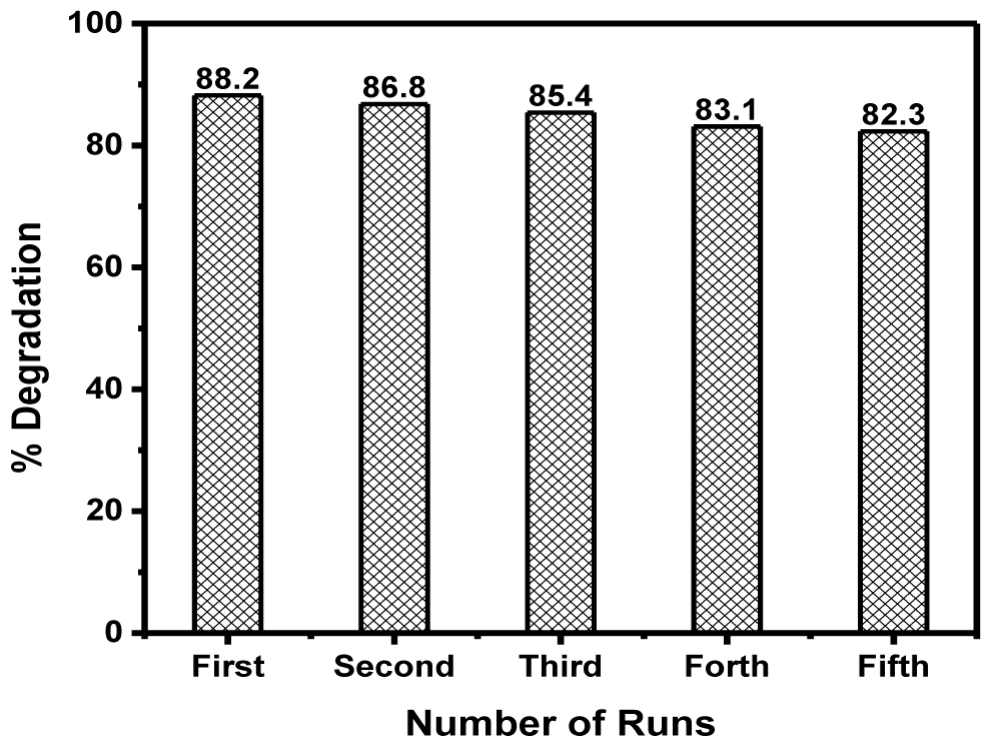
Recyclability test for BiOBr _0.6_I _0.4_ for the removal of 10 mgL ^-1^ MB after 3 h of light illumination at pH 7.0 with 1 gL ^-1^ catalyst dosage.

The recyclability/reusability of a material is key for its practical application. Therefore, the stability of the prepared materials was determined through the recyclability of BiOBr _0.6_I _0.4 _for the removal of MB. The results are displayed in Figure 7. In this study, BiOBr _0.6_I _0.4_ was reused five times. After every cycle, the catalyst was centrifuged, cleaned, and dried for recovery. The degradation efficiency was reduced to 82% from 88%. The slight drop in efficiency of BiOBr _0.6_I _0.4_, even after five cycles, signifies that the BiOBr _z_I _(1-z)_ materials showed remarkable good stability.

***Thermal contribution on the MB photodegradation under solar*.** It is observed that the photocatalytic efficiency was higher under solar irradiation compared to UV light. Unlike the controlled conditions under which the removal of MB under irradiation from UV was done, the removal under solar was performed in open-air that may have introduced other factors that led to the efficiency enhancement. Therefore, an investigation, particularly on the thermal contribution, was done, with other factors like catalyst loading and dye initial concentration kept constant. BiOBr _0.6_I _0.4_ being the best photocatalyst was chosen for the study. An experiment was performed with a set up having four different reaction vessels: two aluminium foil-covered vessels, one with catalyst, and another without and another two uncovered vessels with similar catalyst conditions (with/without) were set up. The temperatures of the four samples were recorded as 27 ℃ before exposure to sunlight. Table S3 in the Extended data ^33^ shows the changes in temperature with irradiation time.

**Figure 8.**
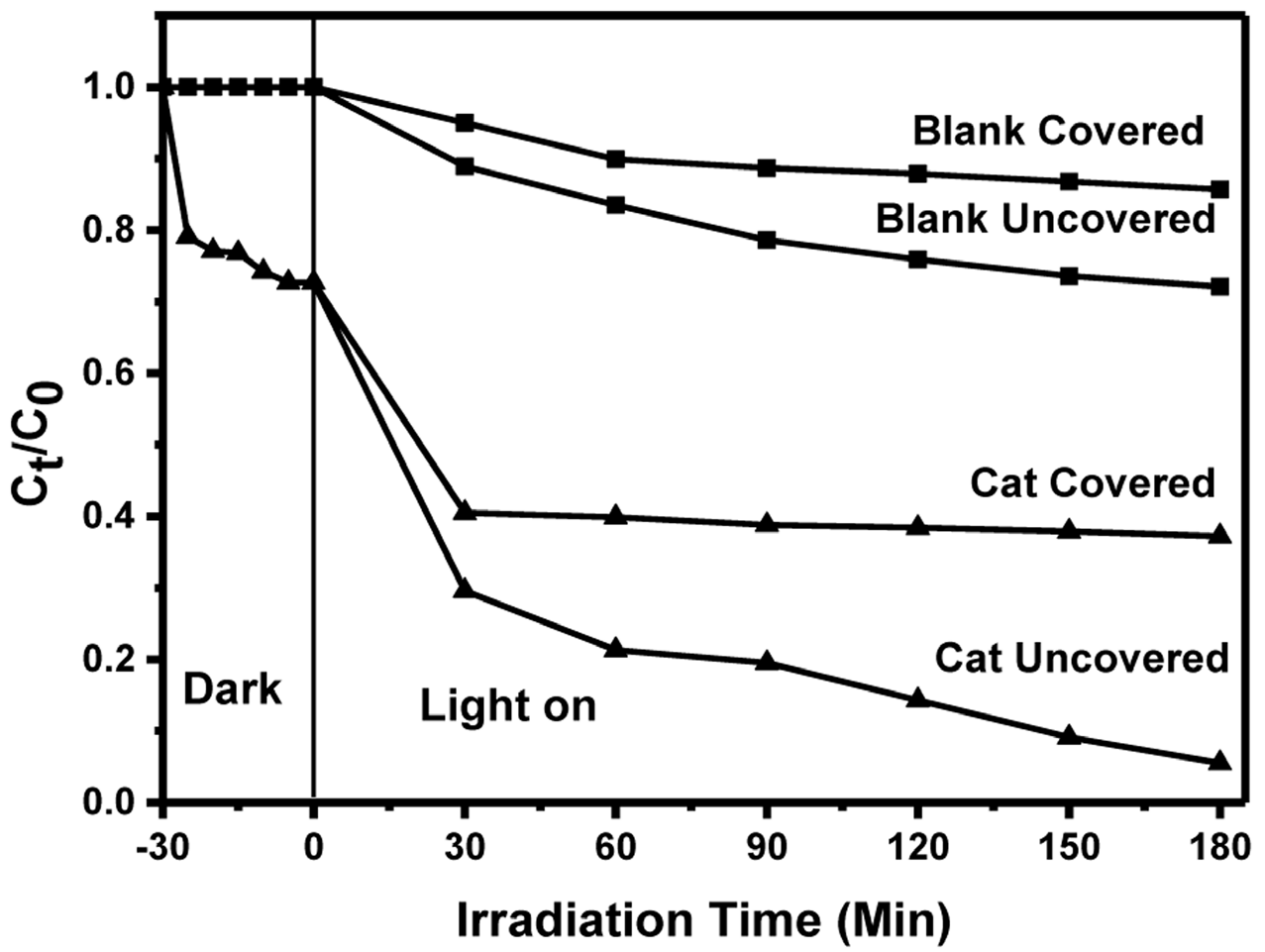
Effects of thermal contribution towards MB degradation under solar irradiation.

For the covered vessels, the temperature increases steadily up to the 150 ^th ^minute whereas for the uncovered vessels, the temperature increases up to the 120 ^th ^minute, with no noticeable change thereafter. The covered vessels recorded the highest temperature due to the build-up of heat that could not escape as the vessels were covered. Further, MB degradation still occurred in the covered vessels, despite the absence of light, which may be attributed to the build-up of heat energy. Figure 8 illustrates the photocatalytic change for MB both from covered and uncovered flasks.

***Effects of temperature*.** Temperature is a relevant parameter since the rate of reaction is temperature dependent. The influence of temperature on the degradation process of MB over BiOBr _0.6_I _0.4_ is shown for the temperature range 30 – 60°C in Figure S8a of the Extended data ^33^. Other parameters were kept constant. It was observed that a rise in temperature caused a gradual increase in degradation efficiency. An increase in temperature led to the enhancement in photooxidation as a result of the increased frequency of molecules collision. Furthermore, when the temperature increases, the reaction competition is increased thereby restraining electron-hole recombination ^37– 39^. Figure S8b displays the Arrhenius plot of k versus T⁻ ^1^ where the activation energy (Ea) of 9.1 × 10⁻ ^1^ J mol⁻ ^1 ^was obtained for the removal of MB. The lower activation energy implies that the removal of MB over BiOBr _0.6_I _0.4_ requires little energy hence making it economical.

***Effect of amount of catalyst on MB removal *.** To determine the influence of the amount of catalyst on the MB removal, a dosage of BiOBr _0.6_I _0.4 _was increased from 0.01 to 0.07 g/L under optimal conditions (10 mg/L MB concentration, pH, 7.0, and 150 min reaction time). The result is shown in the Extended data, Figure S9 ^33^. The removal of MB was increased from 30% to 99% with an increasing amount of dosage from 0.01 g to 0.05 g. The removal was maximum at 0.05 g, which is the optimal catalyst loading. At the optimal dosage, there is maximum availability of the catalyst surface area plus active sites for the production of active radicals under UV-light irradiation. However, the catalyst dosage above 0.06 g results in a decrease in degradation since the screening effect and light scattering tend to become higher ^40– 42^. Additionally, the illumination of key active primary oxidants in photocatalysis will be prevented when the catalyst dosage exceeds the optimum amount hence the degradation efficiency will reduce ^43, 44^. Economically, therefore, optimum catalyst dosage is vital, as less energy would be required for regeneration.

***Effect of pH on photocatalysis*.** The initial pH of a wastewater solution can significantly influence the process of waste treatment since the extent of MB removal is affected by the charge on the photocatalyst surface. The catalyst surface charge depends on the concentration of OH⁻ and H ^+ ^in aqueous solutions ^41^. To establish the role of pH on MB removal, the initial pH of the mixtures were adjusted to 3.0, 5.0, 7.0, 8.0, and 14.0 by adding 1 M HNO _3_ or 1 M NaOH while other conditions were kept constant (0.05 g BiOBr _0.6_I _0.4_ loading and 10 mg/L MB concentration). When the pH values of the mixtures was lower than pH _PZC, _the surface of the catalyst acquires positive charge while if the pH is high, the material acquire negative charge. The results are shown in Figure S10 of the Extended data ^33^. The degradation efficiencies recorded were 47, 28, 86, 78, and 75% for pH values of 3.0, 5.0, 7.0, 8.0, and 14.0, correspondingly.

The high efficiencies at the pH of 7.0, 8.0, and 14.0 can be ascribed to negatively charge BiOBr _0.6_I _0.4 _(pH _PZC _= 5.3)surface. Thus, if the pH of the mixtures were adjusted to the initial pH of 7.0, 8.0, and 14.0, adsorption of the dye on to the positively charged BiOBr _0.6_I _0.4 _surface will be greater due to electrostatic attractions, which enhances the adsorptive process thereby enhancing the degradation efficiencies. On the other hand, adjusting initial pH of the pollutant to 3.0 and 5.0 reduces the degradation efficiency because of the electrostatic repulsion between BiOBr _0.6_I _0.4 _(pH _PZC _= 5.29)surface which has a positive charge and the cationic dye ^41^. Thus, lowering the degradation efficiency of the material.

## Conclusion

The preparation of a series of BiOBr _z_I _(1-z)_ solid solutions via the hydrothermal technique was successful. The crystal structure, morphology, pore size, and surface area were obtained by XRD, SEM, TEM, and BET, respectively. The investigation of the photocatalytic ability of the prepared BiOBr _z_I _(1-z)_ materials was carried out through the removal of MB, additionally the optimization of the operational parameters was also performed. The XRD revealed that the (102) plane shift to smaller diffraction angles with increasing I composition. From the SEM analysis, it was observed that pure BiOBr was made of ultrathin nanoplates with various sizes whereas the morphology of Z = 0.8, 0.6, 0.4, 0.2, and 0.0 was 3-Dimension flower-like structures comprising of nanosheets. The BET results showed that the prepared photocatalysts had low surface areas. The surface characterization showed that the point of zero charge (PZC) of BiOBr, BiOBr _0.8_I _0.2_, BiOBr _0.6_I _0.4_, BiOBr _0.4_I _0.6_, BiOBr _0.2_I _0.8_, and BiOI was found to be 5.27, 5.27, 5.29, 5.31, 5.38, and 5.39, respectively. The percentage degradation efficiency over BiOBr _0.6_I _0.4_ under solar and UV light irradiation was 99 and 88%, respectively. The experimental data was fitted to the first-order kinetics and the R ^2 ^obtained were 0.997, 0.970, 0.963, 0.926, 0.905, and 0.939 for BiOBr, BiOBr _0.8_I _0.2_, BiOBr _0.6_I _0.4_, BiOBr _0.4_I _0.6_, BiOBr _0.2_I _0.8_, and BiOI, correspondingly. The rate constant of BiOBr _0.6_I _0.4_ was three and six times greater than that of individual BiOBr and BiOI, correspondingly. Even after five runs, the material still showed outstanding efficiency and stability. Hence, this study has demonstrated that BiOBr _z_I _(1-z)_ solid solutions synthesized by hydrothermal technique are promising for the degradation of MB in wastewaters. From the linear and non-linear forms of Langmuir-Hinshelwood (L-H) isotherms, nonlinear provided the most flexible curve fitting functionality that prevented the errors brought by various approximations resulting from simple linear regression. The experimental results well fitted the nonlinear and pseudo-first-order model. From the trapping experiment conducted, it was established that the ^•^O _2_^−^ and h ^+^ radicals were the key active species responsible for the removal of MB. Therefore, the use of solid solutions has promised to be a better route for environmental remediation.

## Data availability

### Underlying data

Zenodo: Synthesis of Bismuth Oxyhalide (BiOBr _z_I _(1-z)_) Solid Solutions for Photodegradation of Methylene Dye. http://doi.org/10.5281/zenodo.4987341 ^33^.

This project contains the following underlying data:

- Degradation, scavanger, pH, catalyst dosage, thermal contribution, XRD, SEM TEM, Raman, Point-of-Zero Charge Raw Data.xlsx

### Extended data

Zenodo: Synthesis of Bismuth Oxyhalide (BiOBrzI(1-z)) Solid Solutions for Photodegradation of Methylene Dye. http://doi.org/10.5281/zenodo.4987341 ^33^.

This project contains the following extended data:

- Supporting Information - Figures and Tables.docx

Data are available under the terms of the Creative Commons Attribution 4.0 International license (CC-BY 4.0).
