# Supplementary material for: Synthesis of bismuth oxyhalide (BiOBr zI (1- z)) solid solutions for photodegradation of methylene blue dye
Source: AAS Open Res. 2022 Feb 4;4:43. Originally published 2021 Aug 23. [Version 2] doi: 10.12688/aasopenres.13249.2 (PMC8442118; doi:10.12688/aasopenres.13249.2)
Supplement: Peer review history

# Peer review: Reviewer response for version 2

**DOI:** 10.21956/aasopenres.14470.r29125

**Published:** 2022-02-14

**Authors:** John Mmari Onyari

**Affiliations:** 1 Department of Chemistry, University of Nairobi, Nairobi, Kenya

Thanks. The revisions made are appropriate.

Is the work clearly and accurately presented and does it cite the current literature?

Yes

If applicable, is the statistical analysis and its interpretation appropriate?

Yes

Are all the source data underlying the results available to ensure full reproducibility?

Yes

Is the study design appropriate and is the work technically sound?

Yes

Are the conclusions drawn adequately supported by the results?

Yes

Are sufficient details of methods and analysis provided to allow replication by others?

Yes

Reviewer Expertise:

NA

I confirm that I have read this submission and believe that I have an appropriate level of expertise to confirm that it is of an acceptable scientific standard.

# Peer review: Reviewer response for version 1

**DOI:** 10.21956/aasopenres.14371.r28852

**Published:** 2021-09-22

**Authors:** John Mmari Onyari

**Affiliations:** 1 Department of Chemistry, University of Nairobi, Nairobi, Kenya

This study, involved the synthesis and characterization of Bismuth oxyhalide (BiOBrz I (1-z)) (0 ≤ z ≤ 1) materials. Bismuth oxyhalide was used in the removal of methylene blue (MB) which is a textile waste that is discharged to the environment. The photocatalytic performance of the materials was examined by photodegradation of the MB done under solar and ultraviolet (UV) light irradiation. The presentation of results is well done and the conclusions drawn in the study were adequately supported by the results.

 The manuscript makes a significant contribution to scientific knowledge and my recommendation is “ **Approved with Reservations.”** The paper is to be accepted for indexing after the minor revisions to address issues summarized below. 

 1) Page 4: “However, to the best of the authors’ knowledge, the application of BiOBrz I (1-z) solid solutions in the treatment of methylene blue (MB) dye and comparative studies on the photocatalytic performance of these materials under natural sunlight and UV light has not been reported.“ Include in the literature review and discussion section work done by Ren *et al.* (2013 ^1^) **.**

 2) Page 5, Photodegradation studies: "The set up for the photodegradation under UV lamp and solar is shown in **Figure S1**." Where is Figure S1 shown?

 3) Page 7: "The corresponding bandgap energies of BiOBrz I (1-z) materials were calculated and the result is shown in Table 1". [ … **results are shown** …].

 4) Page 8 Fitting of the adsorption data to adsorption isotherms using nonlinear model fitting procedures ( **Extended data**, Figure S7 ^33^). See also Page 9 etc … As presented in **Table S3** of the **Extended data** ^33  ^Page 10 …. are shown in Figure S7 of the **Extended data** ^33^. Reference is made to “Extended data” several times. Why not just refer to the key finding and give the reference?

 5) Page 10: Label the x-axis in Figure 6.

 6) Page 11: Check the spelling of “forth” in Figure 7 (replace “forth **” **by “fourth”?).

 7) Page 11 last line: "Figure S8b displays the Arrhenius plot of k" - where is Figure S8b shown?

 8) Page 11 Effect of amount of catalyst on MB removal. To determine the influence of the amount of catalyst on the MB removal, a dosage of BiOBr0.6I 0.4 was increased from 0.01 to 0.07 g/L under **optimal conditions** (10 mg/L MB concentration, pH, 7.0, and 150 min reaction time).- indicate the optimal temperature used.

 9) Page 13 References:

 Pavithra KG, Kumar PS, Jaikumar V, **et al.:** etc. - Why is et al., used in this section? Check the Journal guidelines.

 Silveira E, Marques PP, Silva SS, **et al**.:

 Zhang LH, Zhu Y, Lei BR, et al.:

 Drumond Chequer FM, de Oliveira GAR, Anastacio Ferraz ER, **et al**.:

 Sun X, Zhang Y, Li C, **et al**.:

Is the work clearly and accurately presented and does it cite the current literature?

Yes

If applicable, is the statistical analysis and its interpretation appropriate?

Yes

Are all the source data underlying the results available to ensure full reproducibility?

Yes

Is the study design appropriate and is the work technically sound?

Yes

Are the conclusions drawn adequately supported by the results?

Yes

Are sufficient details of methods and analysis provided to allow replication by others?

Yes

Reviewer Expertise:

Development and Utilization of various adsorbent materials for adsorption of dyes and heavy metals

I confirm that I have read this submission and believe that I have an appropriate level of expertise to confirm that it is of an acceptable scientific standard, however I have significant reservations, as outlined above.

## References

[1] : Synthesis of the bismuth oxyhalide solid solutions with tunable band gap and photocatalytic activities. *Dalton Trans* .2013;42(26) : 10.1039/c3dt50498k 9706-12 10.1039/c3dt50498k 23680910

# Response

**Published:** 2021-12-15

**Authors:** Botswana International University of Science Botswana International University of Science

**Affiliations:** Botswana International University of Science and Technology, Botswana

       15 ^th^ December 2021

**Summary of Revisions.**

**Detailed Response to Reviewers' comments**

 All changes are highlighted in “red” in the revised manuscript. Please refer to these changes for more detailed information.

**Reviewers Comments.**

**Reviewer #3:**

**General Comment: APPROVED WITH RESERVATION**

 This study, involved the synthesis and characterization of Bismuth oxyhalide (BiOBr _z_I _(1-z)_) (0 ≤ z ≤ 1) materials. Bismuth oxyhalide was used in the removal of methylene blue (MB) which is a textile waste that is discharged to the environment. The photocatalytic performance of the materials was examined by photodegradation of the MB done under solar and ultraviolet (UV) light irradiation. The presentation of results is well done and the conclusions drawn in the study were adequately supported by the results. The manuscript makes a significant contribution to scientific knowledge and my recommendation is “ **Approved with Reservations.”** The paper is to be accepted for indexing after the minor revisions to address issues summarized below. 

**Comment #1: ** Page 4: “However, to the best of the authors’ knowledge, the application of BiOBr _z_I _(1-z)_ solid solutions in the treatment of methylene blue (MB) dye and comparative studies on the photocatalytic performance of these materials under natural sunlight and UV light has not been reported.“ Include in the literature review and discussion section work done by Ren *et al.* (2013 ^1^ ) **.**

***Response:***

*The authors are very grateful for that important suggestion and the comment.*

***Literature Review***

***BiOM _z_R _1−z_ (M, R = Cl, Br**, **I) **solid solutions **(Ren et al., 2013)** which was the work done by Ren et al has been added on the literature review part as was suggested by the reviewer. The change has been highlighted on the revised manuscript.*

***Discussion***

*From the X-ray diffractometry data, a shift in diffraction peaks to lower diffraction angles was observed in our study. This observation was also reported by Xu et al., and Ren et al. **(Ren et al., 2013)** **. **The work of the Ren et al has been acknowledged as was suggested by the reviewer on the result and discussion part. One reference was added*

***Reference***

*No. 33 Ren, K., Liu, J., Liang, J., Zhang, K., Zheng, X., Luo, H., Huang, Y., Liu, P., & Yu, X. (2013). Synthesis of the bismuth oxyhalide solid solutions with tunable band gap and photocatalytic activities. Dalton Transactions, 42(26), 9706–9712. https://doi.org/10.1039/c3dt50498k*

**Comment #2: **Photodegradation studies: "The set up for the photodegradation under UV lamp and solar is shown in **Figure S1**." Where is Figure S1 shown?

**Response:**

*The Figure S1 is shown on the Extended Data [35]. The change has been made on the revised manuscript and it is highlighted*

***Reference***

*No. 35 R. O. Gembo, O. Aoyi, S. Majoni, A. Etale, S. Odisitse, and C. K. King’ondu, “Synthesis of bismuth oxyhalide (BiOBr _z_I _(1-z)_) solid solutions for photodegradation of methylene blue  dye,” AAS Open Res., vol. 4, p. 43, 2021, doi: 10.12688/aasopenres.13249.1.*

**Comment #3: ** Page 7: "The corresponding bandgap energies of BiOBrzI(1-z) materials were calculated and the result is shown in Table 1". [ … **results are shown** …].

***Response:***

*Thanks to the reviewer for being able to notice the error. The word “is” has been replaced by “are”. The manuscript has been revised to capture the change.*

**Comment #4:** Page 8 Fitting of the adsorption data to adsorption isotherms using nonlinear model fitting procedures ( **Extended data**, Figure S7 ^35^). See also Page 9 etc … As presented in **Table S3** of the **Extended data** ^35  ^Page 10 …. are shown in Figure S7 of the **Extended data** ^35^. Reference is made to “Extended data” several times. Why not just refer to the key finding and give the reference?

**Response:**

*The suggestion is very significant and the authors appreciate it. However, if all the information is included in the manuscript, it would be congested with a lot of figures hence some information has been kept in the Extended Data therefore the references.*

**Comment #5:**  Page 10: Label the x-axis in Figure 6.

**Response:**

*The comment is appreciated. The X – axis has been labelled as “ **Quenching Agent”. **The change is highlighted on the figure.*

**Comment #6:**   Page 11: Check the spelling of “forth” in Figure 7 (replace “forth **” **by “fourth”?).

**Response:**

*The comment is appreciated. The word **“Forth”** is replaced by **“Fourth”** the change has been made on figure 7*

**Comment #7:** Page 11 last line: "Figure S8b displays the Arrhenius plot of k" - where is Figure S8b shown?

**Response:**

*The comment is appreciated. The Figure S8b is shown on the Extended Data [35]. The change has been made on the revised manuscript and it is highlighted*

***Reference***

*No. 35 R. O. Gembo, O. Aoyi, S. Majoni, A. Etale, S. Odisitse, and C. K. King’ondu, “Synthesis of bismuth oxyhalide (BiOBr _z_I _(1-z)_) solid solutions for photodegradation of methylene blue  dye,” AAS Open Res., vol. 4, p. 43, 2021, doi: 10.12688/aasopenres.13249.1.*

**Comment #8:** Page 11 Effect of amount of catalyst on MB removal. To determine the influence of the amount of catalyst on the MB removal, a dosage of BiOBr _0.6_I _0.4_ was increased from 0.01 to 0.07 g/L under **optimal conditions** (10 mg/L MB concentration, pH, 7.0, and 150 min reaction time).- indicate the optimal temperature used.

**Response:**

*The comment is appreciated. The optimal temperature is **30** **± 2 ℃***

**Comment #9:**  Page 13 References:

 Pavithra KG, Kumar PS, Jaikumar V, **et al.:** etc. - Why is et al., used in this section? Check the Journal guidelines.

 Silveira E, Marques PP, Silva SS, **et al**.:

 Zhang LH, Zhu Y, Lei BR, et al.:

 Drumond Chequer FM, de Oliveira GAR, Anastacio Ferraz ER, **et al**.:

 Sun X, Zhang Y, Li C, **et al**.:

**Response:**

*The authors would to commend the reviewer such an important question. Nonetheless,  The original document includes all the authors in the references. However, the references have the et al because that’s the AAS guideline on presenting the references.*

# Peer review: Reviewer response for version 1

**DOI:** 10.21956/aasopenres.14371.r28856

**Published:** 2021-09-14

**Authors:** Elizabeth N. Ndunda

**Affiliations:** 1 Department of Physical Sciences, School of Pure and Applied Sciences, Machakos University, Machakos, Kenya

Environmental pollution is becoming a global challenge and studies on remediation techniques for these environmental pollutants are timely. The current manuscript clearly details the degradation of methylene blue dye, which is found in textile waste, using bismuth oxyhalide. The manuscript is well written, with the following minor suggestions:

1. The title needs to be revised to capture methylene blue dye as used in text.
2. The authors need to mention if they tried the catalyst on real samples.
3. Besides MB being a textile waste, the authors should further mention any effects that MB might have on humans, animals and the environment.

Is the work clearly and accurately presented and does it cite the current literature?

Yes

If applicable, is the statistical analysis and its interpretation appropriate?

Yes

Are all the source data underlying the results available to ensure full reproducibility?

Yes

Is the study design appropriate and is the work technically sound?

Yes

Are the conclusions drawn adequately supported by the results?

Yes

Are sufficient details of methods and analysis provided to allow replication by others?

Yes

Reviewer Expertise:

fabrication of sensor, synthesis of molecularly imprinted polymers for analytical applications.

I confirm that I have read this submission and believe that I have an appropriate level of expertise to confirm that it is of an acceptable scientific standard.

# Response

**Published:** 2021-12-15

**Authors:** Botswana International University of Science Botswana International University of Science

**Affiliations:** Botswana International University of Science and Technology, Botswana

       15 ^th^ December 2021

**Summary of Revisions.**

**Detailed Response to Reviewers' comments**

 All changes are highlighted in “red” in the revised manuscript. Please refer to these changes for more detailed information.

**Reviewers Comments.**

**Reviewer #2**:

**General Comment: APPROVED**

 Environmental pollution is becoming a global challenge and studies on remediation techniques for these environmental pollutants are timely. The current manuscript clearly details the degradation of methylene blue dye, which is found in textile waste, using bismuth oxyhalide. The manuscript is well written, with the following minor suggestions:

**Comment #1: **The title needs to be revised to capture methylene blue dye as used in text.

***Response: **The authors acknowledge the reviewer for such an important observation about the title **“**Synthesis of bismuth oxyhalide (BiOBr _z_I _(1-z)_) solid solutions for photodegradation of methylene dye”. The title has been revised as suggested. The change has been highlighted on the revised manuscript.*

***New title: **Synthesis of bismuth oxyhalide (BiOBr _z_I _(1-z)_) solid solutions for photodegradation of methylene blue dye*

**Comment #2: **The authors need to mention if they tried the catalyst on real samples.

**Response:** *Thanks to the reviewer for such a vital concern. However, the catalyst was not tried on the real samples because there is no textile industry around the university where the study was carried out, therefore the authors resorted for synthetic textile waste.*

**Comment #3: **Besides MB being a textile waste, the authors should further mention any effects that MB might have on humans, animals and the environment.

***Response: **Thanks to the reviewer for that significant question on whether the dye has effect on both human and animals.*

***Added paragraph on the revised manuscript:** … Methylene blue dye if not monitored can adversely affect human beings and animals by causing eye burns which is a permanent health damage. Additionally, the MB can cause painful micturition, nausea, increase in heart rate, quadriplegia disorders in breathing, quadriplegia, cyanosis, mental confusion, jaundice, vomiting, tissue necrosis, and methemoglobinemia. **(Ramírez-Aparicio et al., 2021)** **.***

*The reference below was added in response to the comment.*

***Reference***

*No. 34 Ramírez-Aparicio, J., Samaniego-Benítez, J. E., Murillo-Tovar, M. A., Benítez-Benítez, J. L., Muñoz-Sandoval, E., & García-Betancourt, M. L. (2021). Removal and surface photocatalytic degradation of methylene blue on carbon nanostructures. Diamond and Related Materials, 119(July), 108544. https://doi.org/10.1016/j.diamond.2021.108544.*

# Peer review: Reviewer response for version 1

**DOI:** 10.21956/aasopenres.14371.r28854

**Published:** 2021-09-08

**Authors:** Ndzondelelo Bingwa

**Affiliations:** 1 Center for Synthesis and Catalysis, Department of Chemical Sciences, University of Johannesburg, Johannesburg, South Africa

The authors describe synthesis of bismuth oxyhalides and their application in photocatalytic degradation of an industrial (textile) pollutant, methylene blue. They further studied the kinetics of adsorption using the Langmuir-based kinetic model. The results are well presented. The structure of the work is commendable. However, there are a few minor issues that need to be taken care of.

1. The product(s) of degradation must be stated in the abstract. Otherwise, the reader might think that the only process taking place is removal by adsorption.
2. The magnification on the TEM images is too low. It would be great if the images are taken at higher magnification. This will allow the determination of interplanner spacing.
3. The catalytic recyclability and reusability is a study can be reported in a better manner. Comparing conversions at the end of the reaction or at a certain reaction time might be deceiving. It is always advisable to study catalyst reusability with kinetics. In this way, it is easy to see if the catalyst is deactivating by looking at the reaction rates rather than conversion at time t.

Is the work clearly and accurately presented and does it cite the current literature?

Yes

If applicable, is the statistical analysis and its interpretation appropriate?

Yes

Are all the source data underlying the results available to ensure full reproducibility?

Yes

Is the study design appropriate and is the work technically sound?

Yes

Are the conclusions drawn adequately supported by the results?

Yes

Are sufficient details of methods and analysis provided to allow replication by others?

Yes

Reviewer Expertise:

Heterogeneous catalysis, material synthesis, photocatalysis, nanomaterials for catalysis

I confirm that I have read this submission and believe that I have an appropriate level of expertise to confirm that it is of an acceptable scientific standard.

# Response

**Published:** 2021-12-15

**Authors:** Botswana International University of Science Botswana International University of Science

**Affiliations:** Botswana International University of Science and Technology, Botswana

15 ^th^ December 2021

**Summary of Revisions.**

**Detailed Response to Reviewers' comments**

 All changes are highlighted in “red” in the revised manuscript. Please refer to these changes for more detailed information.

**Reviewers Comments.**

**Reviewer #1**:

**General Comment: APPROVED**

 The authors describe synthesis of bismuth oxyhalides and their application in photocatalytic degradation of an industrial (textile) pollutant, methylene blue. They further studied the kinetics of adsorption using the Langmuir-based kinetic model. The results are well presented. The structure of the work is commendable. However, there are a few minor issues that need to be taken care of.

**Comment #1: **The product(s) of degradation must be stated in the abstract. Otherwise, the reader might think that the only process taking place is removal by adsorption.

**Response:** *Thanks to the reviewer for that vital comment. Nevertheless, the degradation products were not analyzed as it requires LC-MS which is not available at our university, therefore the analysis was not possible. Nevertheless, the degradation was monitored through color reduction.*

**Comment #2: **The magnification on the TEM images is too low. It would be great if the images are taken at higher magnification. This will allow the determination of interplanar spacing.

**Response:** *The authors thank the reviewer for this excellent suggestion. It is true that the magnification applied was low to reveal the interplanar spacing. However, the interplanar spacing can be determined by high resolution transmission electron microscope unlike in this work where the materials were analyzed using transmission electron microscope which only gives the micrographs.*

**Comment #3: **The catalytic recyclability and reusability is a study can be reported in a better manner. Comparing conversions at the end of the reaction or at a certain reaction time might be deceiving. It is always advisable to study catalyst reusability with kinetics. In this way, it is easy to see if the catalyst is deactivating by looking at the reaction rates rather than conversion at time t.

**Response:** *T he authors thank the reviewer for this excellent suggestion that provide more insight and evidence on the stability of the prepared materials, unfortunately a stability with kinetics was not studied as the authors only did a study to establish the stability of the prepared photocatalysts. The cyclic voltammetry was not performed to establish whether the catalyst was deactivating or not.*
